# Respiratory infections and asthma exacerbations in children: Regional insights into the overlooked role of atypical pathogens in Mexico

**DOI:** 10.1016/j.jacig.2025.100597

**Published:** 2025-10-31

**Authors:** Victor Gonzalez-Uribe, Carlos Andres Gomez-Nuñez, Ricardo Martinez-Tenopala, Jimena Prieto-Gomez, Luis Angel Hernandez-Zarate, Alejandra Revelles-Martinez, Maria Julia Rendon-Salazar, Tamara Hernandez-Hernandez, Blanca Estela del Rio-Navarro, Arturo Berber, Christian Rodrigo Alcocer-Arreguin, Paola de Baro Alvarez, Zaira S. Mojica-Gonzalez

**Affiliations:** a AlergiaMx, Benito Juarez, Mexico City, Mexico; b Facultad Mexicana de Medicina, Universidad La Salle Mexico, Tlalpan, Mexico City, Mexico; c Hospital Infantil de Mexico Federico Gomez, Cuauhtemoc, Mexico City, Mexico; d Pediatric Allergy & Clinical Immunology Laboratory, Hospital Infantil de México Federico Gomez, Cuauhtemoc, Mexico City, Mexico; e Sin Alergia GDL, Guadalajara, Jalisco, Mexico; f Pathology & Immunohistochemistry Department, Hospital General de Mexico Dr. Eduardo Liceaga, Cuauhtemoc, Mexico City, Mexico

**Keywords:** Asthma exacerbation, respiratory infections, wheezing, *Bordetella pertussis*, Latin America, atypical pathogens, epidemiology

## Abstract

**Background:**

Respiratory infections are the leading cause of asthma exacerbations in children, yet data from Latin America remain limited, especially in the postpandemic context. Understanding the respiratory pathogen landscape in pediatric patients with wheezing episodes may inform regional management strategies.

**Objective:**

We sought to characterize the prevalence of respiratory pathogens, including atypical viruses and bacteria, in children with asthma or recurrent wheezing in 2 distinct regions of Mexico.

**Methods:**

We conducted a retrospective study of 432 children (aged 2-17 years) with a history of asthma or wheezing and current lower respiratory tract symptoms. Nasopharyngeal swabs were collected between January 2021 and September 2022 at tertiary centers in Mexico City and Oaxaca. Multiplex PCR detected 16 viruses and 3 atypical bacteria. Severe acute respiratory syndrome coronavirus 2 was tested separately by RT-PCR. Statistical comparisons were performed by region and age group.

**Results:**

Seasonal coronaviruses were prominent, with coronavirus 229E being more prevalent in Mexico City (24.6%) than in Oaxaca (10.2%; *P* < .001). Influenza A (5.6% vs 14.3%, *P* = .002), parainfluenza virus type 2 (6.3% vs 16.3%, *P* < .001), and *Bordetella pertussis* were more prevalent in Oaxaca (12.2%) than in Mexico City (2.8%, *P* < .001). Coinfections occurred in 30.3% of patients and were more frequent in Oaxaca (38.8% vs 26.0%, *P* = .008).

**Conclusions:**

Atypical viruses and *B pertussis* were frequently detected in children with wheezing in postpandemic Mexico. Findings highlight regional disparities in pathogen circulation and the need for enhanced surveillance and vaccination strategies across Latin America.

Asthma is a common chronic inflammatory airway disease characterized by reversible airflow limitation and bronchial hyperresponsiveness, affecting more than 8% of children and adults worldwide.^1^ Exacerbations of asthma are a major cause of morbidity, often leading to emergency department visits, hospitalizations, and even death in severe cases.^2^ The pathophysiology of asthma involves chronic airway inflammation and immune dysregulation, which can prime the airways for heightened responses to environmental triggers. Among such triggers, respiratory viral infections have emerged as the predominant instigators of asthma exacerbations.^3^, ^4^, ^5^

A large body of evidence indicates that most asthma exacerbations are precipitated by respiratory viruses. Epidemiologic studies using sensitive PCR-based virus detection report that viral infections can be identified in 80% to 95% of children experiencing acute wheezing or asthma exacerbation, and in roughly 80% of adult asthma exacerbations.^6^, ^7^, ^8^ Among these, human rhinovirus (RV)^9^ is the single most frequent culprit in both pediatric and adult exacerbations.^10^ RV (especially species A and C) is detected in approximately 60% to 80% of children with asthma attacks requiring acute care^1^ and in a large proportion of adult cases as well.^11^ Other respiratory viruses, including respiratory syncytial virus, influenza, parainfluenza, adenovirus, human metapneumovirus (hMPV), and bocavirus, also contribute to exacerbations but to a lesser extent.^10^

Despite the global recognition of viruses as key asthma triggers, there is a relative dearth of high-quality epidemiological data from Latin America exploring these associations. Most large cohort and surveillance studies on virus-induced asthma exacerbations have been conducted in North America, Europe, and Asia, whereas Latin American populations remain underrepresented.^12^, ^13^, ^14^ The available evidence suggests that viral triggers in Latin America are broadly similar to those elsewhere; for example, a study in Mexico City found respiratory viruses in approximately 60% of children younger than 15 years who were hospitalized with acute asthma or respiratory distress, with RV identified as the most frequent etiologic agent.^12^ Latin America lacks extensive, multicenter data on the prevalence and impact of viral infections in asthma exacerbations. This gap is particularly important given the diverse climates and health care settings across the region, which might influence viral transmission and asthma management. The postpandemic shifts in virus circulation further complicate the picture, and their implications for Latin American patients with asthma are not well documented. In summary, there is a crucial need for more comprehensive epidemiologic research in Latin America to characterize viral triggers of asthma exacerbations. Such data would help inform region-specific strategies for asthma prevention and acute care, for instance, guiding vaccination policies (eg, influenza or future RSV vaccines), antiviral interventions, and public health measures to mitigate asthma crises precipitated by viral epidemics.

## Methods

### Study design and population

This retrospective observational study included 432 children older than 2 years (up to age 17 years) with acute respiratory symptoms and a history of wheezing or asthma. Patients were evaluated between January 2021 and September 2022 at 2 tertiary care centers in Mexico (Mexico City and Oaxaca), predominantly urban and semiurban populations; both hospitals are referral centers serving primarily uninsured populations. Inclusion criteria required the presence of lower acute respiratory tract symptoms (such as cough, difficulty breathing, or wheezing), together with either a previous physician-documented diagnosis of asthma or at least 2 documented episodes of wheezing recorded by a health care provider. Parental or self-report alone, without medical documentation, was not considered sufficient. Clinical data, including patient demographics and medical history, were obtained from electronic medical records. Information on prescription of controller medications was inconsistently available and therefore not included in the primary analysis. The study protocol was approved by the institutional review boards of both participating centers, with a waiver of informed consent due to the retrospective design. Any patient with chronic respiratory conditions other than asthma, immunodeficiency, or incomplete clinical data was excluded from the study.

### Laboratory testing

Nasopharyngeal swab specimens were collected from each patient at the time of evaluation. Pathogen detection was performed using the FilmArray Respiratory Panel (BioFire Diagnostics, Salt Lake City, Utah), a multiplex real-time PCR system that targets 16 respiratory viruses and 3 atypical bacteria. The viral targets included common respiratory pathogens such as respiratory syncytial virus, influenza A and B viruses, parainfluenza virus types 1 to 4, adenovirus, hMPV, RV/enterovirus, and common coronaviruses (229E, NL63, OC43, HKU1). The bacterial targets included *Mycoplasma pneumoniae*, *Chlamydophila pneumoniae*, and *Bordetella pertussis*. In addition, all specimens were tested for severe acute respiratory syndrome coronavirus 2 (SARS-CoV-2) using a separate reverse transcription PCR (RT-PCR) assay from Thermo Fisher Scientific (Waltham, Mass), following the manufacturer’s protocol. During the coronavirus disease 2019 (COVID-19) pandemic, multiplex PCR testing was part of institutional protocols for all patients presenting with acute respiratory symptoms; therefore, all the children included in the present study were tested following the standard care procedures. All assays were performed according to standard laboratory procedures with appropriate quality control measures.

### Statistical analysis

Descriptive statistics were used to summarize patient characteristics and pathogen detection frequencies. Categorical variables (eg, pathogen detection rates between the 2 sites) were compared using chi-square tests or Fisher exact tests, and continuous variables (eg, age) were compared using Student *t* tests or Mann-Whitney *U* tests for non–normally distributed data. Coinfection, defined as the detection of 2 or more pathogens in the same sample, was assessed for frequency and compared between sites. Multivariable logistic regression analysis was performed to identify independent predictors of a positive pathogen test result, adjusting for potential confounders (such as age, sex, and site). All statistical tests were 2-tailed, with *P* less than .05 considered statistically significant. Data analysis was performed using IBM SPSS Statistics, version 26 (IBM Corp, Armonk, NY).

## Results

A total of 432 pediatric patients with a history of asthma or recurrent wheezing and acute respiratory symptoms were included in this study: 285 from Mexico City and 147 from Oaxaca. The mean age was comparable across sites, with a balanced distribution among the 2- to 5-, 6- to 11-, and 12- to 17-year-old groups. All included patients had at least 1 respiratory pathogen detected through PCR analysis.

### Pathogen prevalence by site

Respiratory pathogens were widely detected in both regions, though with significant variation in the distribution of specific viruses and bacteria (Table I). Coronavirus 229E was significantly more prevalent in Mexico City (24.6%) than in Oaxaca (10.2%) (*P* < .001), suggesting local differences in viral circulation. Other seasonal coronaviruses such as OC43 (12.3% in Mexico City vs 5.4% in Oaxaca, *P* = .024) followed a similar pattern, whereas NL63 and HKU1 showed no significant site differences.

**TABLE I:** Prevalence∗ of respiratory pathogens by site (Mexico City vs Oaxaca)

Pathogen	Mexico City (n = 285)	Oaxaca (n = 147)	*P* value†
Adenovirus	15 (5.3)	7 (4.8)	.822
Coronavirus 229E	70 (24.6)	15 (10.2)	<.001
Coronavirus HKU1	25 (8.8)	12 (8.2)	.83
Coronavirus OC43	35 (12.3)	8 (5.4)	.024
Coronavirus NL63	12 (4.2)	8 (5.4)	.564
hMPV	37 (13.0)	17 (11.6)	.673
Human RV/enterovirus	18 (6.3)	7 (4.8)	.512
Influenza A	16 (5.6)	21 (14.3)	.002
Influenza A (H1)	16 (5.6)	6 (4.1)	.492
Influenza A (H1N1)pdm09	12 (4.2)	0 (0.0)	.01
Influenza A (H3)	12 (4.2)	6 (4.1)	.949
Influenza B	7 (2.5)	16 (10.9)	<.001
Parainfluenza 1	5 (1.8)	4 (2.7)	.497
Parainfluenza 2	18 (6.3)	24 (16.3)	<.001
Parainfluenza 3	15 (5.3)	10 (6.8)	.516
Parainfluenza 4	6 (2.1)	4 (2.7)	.74
SARS-CoV-2	32 (11.2)	28 (19.0)	.026
*Bordetella pertussis*	8 (2.8)	18 (12.2)	<.001
*Mycoplasma pneumoniae*	0 (0.0)	1 (0.7)	.34
*Chlamydophila pneumoniae*	5 (1.8)	0 (0.0)	.171

∗ Values are number of positive cases (percentage of total in parentheses).

† *P* values calculated using χ^2^ or Fisher exact test, as appropriate.

In contrast, certain pathogens were disproportionately more frequent in Oaxaca. Influenza A (14.3% vs 5.6%, *P* = .002) and influenza B (10.9% vs 2.5%, *P* < .001) were significantly more common in the Oaxaca cohort. Similarly, parainfluenza virus (PIV) type 2 (PIV2) showed marked disparity (16.3% in Oaxaca vs 6.3% in Mexico City, *P* < .001), as did SARS-CoV-2 (19.0% vs 11.2%, *P* = .026). The most striking difference was observed for *B pertussis*, with 12.2% of patients testing positive in Oaxaca compared with just 2.8% in Mexico City (*P* < .001).

### Pathogen distribution by age group

When stratified by age group (Table II), most viral pathogens demonstrated consistent distribution across the 2- to 5-, 6- to 11, and 12- to 17-year-old age ranges. For example, detection rates for adenovirus, coronaviruses, and metapneumovirus remained relatively stable across all groups. Interestingly, SARS-CoV-2 positivity increased with age, from 7.7% in the youngest group to 18.8% in adolescents. A similar trend was noted for RV/enterovirus and influenza A H1N1, which were slightly more frequent in older children.

**TABLE II:** Prevalence∗ of respiratory pathogens by age group†

Pathogen	2-5 y (n = 169)	6-11 y (n = 133)	12-17 y (n = 117)
Adenovirus	9 (5.3)	7 (5.3)	6 (5.1)
Coronavirus 229E	33 (19.5)	27 (20.3)	22 (18.8)
Coronavirus HKU1	17 (10.1)	8 (6.0)	9 (7.7)
Coronavirus OC43	15 (8.9)	7 (5.3)	9 (7.7)
Coronavirus NL63	10 (5.9)	4 (3.0)	3 (2.6)
hMPV	26 (15.4)	12 (9.0)	12 (10.3)
Human RV/enterovirus	8 (4.7)	9 (6.8)	10 (8.5)
Influenza A	12 (7.1)	9 (6.8)	7 (6.0)
Influenza A (H1)	6 (3.6)	9 (6.8)	7 (6.0)
Influenza A (H1N1)pdm09	3 (1.8)	4 (3.0)	5 (4.3)
Influenza A (H3)	6 (3.6)	8 (6.0)	2 (1.7)
Influenza B	11 (6.5)	13 (9.8)	6 (5.1)
Parainfluenza 1	3 (1.8)	2 (1.5)	1 (0.9)
Parainfluenza 2	17 (10.1)	16 (12.0)	8 (6.8)
Parainfluenza 3	10 (5.9)	4 (3.0)	7 (6.0)
Parainfluenza 4	5 (3.0)	2 (1.5)	1 (0.9)
SARS-CoV-2	13 (7.7)	22 (16.5)	22 (18.8)
*Bordetella pertussis*	6 (3.6)	4 (3.0)	5 (4.3)
*Mycoplasma pneumoniae*	0 (0.0)	0 (0.0)	1 (0.9)
*Chlamydophila pneumoniae*	1 (0.6)	2 (1.5)	0 (0.0)

The total number of participants in Table II (n = 419) differs from that in Table I because age-stratified analyses included only children with complete age data within the 2-17-y-old range (13 patients excluded).

∗ Values are number of positive cases (percentage of total in parentheses).

† Age groups: 2-5 y, 6-11 y, 12-17 y.

Bacterial pathogens such as *B pertussis* were evenly distributed across age groups, although slightly higher rates were observed in the 12- to 17-year-old group (4.3%). *M pneumoniae* was detected only in 1 adolescent from Oaxaca, whereas *C pneumoniae* was infrequently found and limited to younger age groups.

### Coinfection patterns

Coinfections, defined as the simultaneous detection of 2 or more pathogens in a single sample, were common, occurring in 30.3% of all patients (Table III). However, this rate differed significantly between sites: 38.8% in Oaxaca versus 26.0% in Mexico City (*P* = .008). Most coinfections involved 2 viruses, detected in 56 patients from Mexico City and 39 from Oaxaca. Triple infections were less frequent and consisted of 2 viruses plus 1 bacterium: 1 case in Mexico City (*B pertussis*) and 7 in Oaxaca (6 *B* pertussis and 1 *M pneumoniae*). Viral-viral combinations were most often associated with seasonal coronaviruses (eg, OC43 and 229E), metapneumovirus, and RV/enterovirus. Viral-bacterial coinfections predominantly involved *B pertussis*. The clinical relevance of these codetections especially in children presenting with wheezing or lower respiratory tract symptoms warrants further investigation, because multiple concurrent pathogens may exacerbate inflammation and increase symptom burden.

**TABLE III:** Summary of coinfection patterns

Metric	Mexico City	Oaxaca	Total
Total patients	285	147	432
Coinfected patients	57	46	103
Coinfection∗ rate	20.0%	31.3%	23.8%
Triple infections	1	7	8

∗ Coinfection defined as detection of ≥2 pathogens in the same sample.

In summary, Oaxaca exhibited significantly higher frequencies of influenza A and B, parainfluenza 2, SARS-CoV-2, and *B pertussis* compared with Mexico City. Meanwhile, Mexico City showed higher detection rates of coronavirus 229E and OC43.

## Discussion

Our study revealed viral detection patterns broadly consistent with other large studies, but with notably high rates of some typically milder pathogens. RV/enterovirus and influenza were common, as in other pediatric influenza-like illness series, but we also found substantial circulation of seasonal coronaviruses (especially CoV-229E), hMPV, and parainfluenza viruses. For example, CoV-229E was detected in 24.6% (70 of 285) of samples in Mexico City and 10.2% (15 of 147) in Oaxaca, even though systematic reviews report 229E as one of the least frequently detected seasonal coronaviruses in children.^15^ Likewise, PIV2 was found in 6.3% (18 of 285) of Mexico City samples and 16.3% (24 of 147) in of Oaxaca samples; in comparison, PIV1 was detected in 1.8% and 2.7%, and PIV3 in 5.3% (15 of 285) and 6.8% (10 of 147), respectively. Global surveillance shows that PIV2 is generally much less common than PIV1 or PIV3 and often causes milder croup-like illness.^16^ hMPV appeared in 13.0% (37 of 285) of Mexico City patients and 11.6% (17 of 147) of Oaxaca patients, in line with previous pediatric studies reporting hMPV in roughly 5% to 7% of acute respiratory infection cases.^9^ Overall, our virus profile mirrors global pediatric cohorts: RV/enterovirus and influenza typically dominate, whereas “atypical” viruses (respiratory adenoviruses, bocaviruses, seasonal coronaviruses, hMPV, and PIV2/4) occur at lower but still appreciable rates.^9^^,^^15^ This study spanned the period from January 2021 to September 2022; however, we did not stratify pathogen detection by season. This represents a limitation, because seasonal variation in respiratory virus circulation is well documented. Another limitation is the absence of a comparator group of children without asthma with respiratory symptoms during the same period, which restricts our ability to determine whether the pathogen distribution observed was unique to asthmatic children or simply reflected community circulation patterns, particularly in the context of post-COVID shifts in viral epidemiology. Future studies should incorporate temporal analyses to identify peak circulation periods and include both asthmatic and nonasthmatic cohorts to better delineate the specific contribution of respiratory pathogens to asthma exacerbations and to optimize the timing of preventive interventions such as vaccination.

SARS-CoV-2 detection in our cohort also reflects known pediatric trends. By early 2022, many children remained seronegative: one meta-analysis found that only approximately 30% to 50% of children globally had evidence of SARS-CoV-2 infection by late 2021.^17^ Reported pediatric case rates varied widely, from approximately 1% to 22% in infants across different countries,^18^, ^19^, ^20^ in part because infections were often asymptomatic or untested.^21^ In our sample, SARS-CoV-2 was detected in 11.2% of children in Mexico City (32 of 285) and 19.0% in Oaxaca (28 of 147), reflecting high community transmission during the Omicron era. These proportions exceed the pre-Omicron seroprevalence estimates, consistent with the sharp rise in pediatric infections during that wave. Children generally experience milder COVID-19, but Omicron significantly increased pediatric case counts and hospitalizations worldwide.^21^ Our findings therefore align with global data showing substantial SARS-CoV-2 circulation in children by 2022, especially after Omicron, even though many pediatric infections remain underrecognized.^22^^,^^23^

In contrast to viral infections, *B pertussis* results highlight vaccine- and socioeconomic-driven disparities. *B pertussis* was detected in 12.2% of children in Oaxaca (18 of 147) and 2.8% in Mexico City (8 of 285). Oaxaca is one of Mexico’s most impoverished, rural states with a large indigenous population and limited health care access.^24^, ^25^, ^26^ These factors likely contribute to low immunization uptake and diagnostic delays. Mexico’s *B pertussis* history has included endemic transmission and periodic outbreaks: reported cases rose from approximately 50/y in 2000 to more than 550 in 2009,^27^, ^28^, ^29^ and large surges occurred in the period 2012 to 2016.^30^ A recent national study found that vaccine effectiveness remains high (>95%) but that coverage falls off with each dose, and booster doses are often incomplete.^30^ Likewise, Latin American reviews note that 3-dose diphtheria-pertussis-tetanus vaccine coverage is highly variable and rarely 90% or higher.^31^ The Pan American Health Organization has warned of *B pertussis* resurgence as COVID-19 disrupted routine immunizations and created “immunity gaps.” Globally, diphtheria-tetanus-pertussis coverage fell from 86% (2019) to 81% (2021) due to pandemic disruptions.^32^ In Mexico, vaccination campaigns were similarly affected, and marginalized communities saw larger declines. Taken together, the high *B pertussis* rate in Oaxaca likely reflects both preexisting low coverage in rural/indigenous regions and additional lapses in booster programs or catch-up campaigns post-COVID. In contrast, Mexico City’s higher baseline coverage and access help explain its lower *B pertussis* positivity. These findings echo Latin American patterns: countries with uneven vaccine coverage (and recent declines) have seen *B pertussis* outbreaks in infants, prompting calls for strengthened immunization (including maternal Tdap) and surveillance.^31^^,^^33^

### Conclusion

This study offers a comprehensive view of the respiratory pathogens identified in Mexican children presenting with symptoms of asthma exacerbation, wheezing, or lower respiratory tract distress during the postpandemic period. Beyond the expected circulation of influenza and RV, we observed a notable presence of respiratory pathogens not classically linked to asthma exacerbations, including seasonal coronavirus 229E, parainfluenza virus type 2, and hMPV. These findings suggest that a broader range of respiratory viruses may contribute to lower airway inflammation and wheezing episodes in pediatric populations, particularly in the context of post-COVID immunologic shifts.

The detection of *B pertussis*—especially its high prevalence in the Oaxaca cohort—further expands the differential for nonviral triggers of wheezing and underscores the clinical importance of considering atypical bacterial pathogens in regions with known gaps in immunization and health care access.

Together, these results highlight the evolving landscape of infectious contributors to pediatric respiratory morbidity. They emphasize the need to expand diagnostic and surveillance efforts beyond traditional pathogens, adapt asthma management to regional microbial epidemiology, and prioritize public health strategies that reinforce equitable vaccine access and early recognition of atypical causes of wheezing and asthma-like symptoms in children.

Ethics statement: This study was conducted in accordance with the ethical principles of the Declaration of Helsinki. The research protocol was reviewed and approved by the institutional review boards of both participating centers. Because of the retrospective nature of the study and the use of deidentified clinical data, the requirement for informed consent was waived by the ethics committees.

## Disclosure statement

This study did not receive any specific grant from funding agencies in the public, commercial, or not-for-profit sectors.

Disclosure of potential conflict of interest: The authors declare that they have no relevant conflicts of interest.
